# LRIG1 is a positive prognostic marker in Merkel cell carcinoma and Merkel cell carcinoma expresses epithelial stem cell markers

**DOI:** 10.1007/s00428-021-03158-7

**Published:** 2021-07-31

**Authors:** Benjamin Sundqvist, Harri Sihto, Maria von Willebrand, Tom Böhling, Virve Koljonen

**Affiliations:** 1 Department of Pathology, Haartman Institute, University of Helsinki, P.O. Box 21, 00014 Helsinki, Finland; 2 Department of Plastic Surgery, University of Helsinki and Helsinki University Hospital, Helsinki, Finland

**Keywords:** Merkel cell carcinoma, Stem cell, Hair follicle, LRIG1, Immunohistochemistry

## Abstract

Merkel cell carcinoma (MCC) is a rare and aggressive neuroendocrine malignancy of the skin. The cell of origin of MCC is thus far unknown and proposed cells of origin include Merkel cells, pro-/pre- or pre-B cells, epithelial stem cells, and dermal stem cells. In this study, we aimed to shed further light on the possibility that a subset of MCC tumors arise from epithelial stem cells of the skin by examining the expression of hair follicle and epidermal stem cell markers in MCC and normal human skin. We also aimed to elucidate any correlation between the expression of these markers and tumor Merkel cell polyomavirus (MCPyV) status or other clinicopathological characteristics or patient survival. Expression of CK19, SOX9, LGR5, and LRIG1 in MCC and normal human skin was studied by immunohistochemistry, and the staining patterns or intensities were statistically correlated with patient, tumor, MCPyV, and survival parameters. In a cohort of 137 cases of MCC, we observed dot-like immunoexpression of CK19 in 30 cases (22.1%) and homogeneous expression in 103 cases (75.7%). We also observed positive immunoexpression of SOX9 in 21 cases (15.3%), LGR5 in 118 cases (86.1%), and LRIG1 in 117 cases (86.0%). Immunoexpression of LRIG1 was found to correlate with better overall and MCC-specific survival. We observed frequent immunoexpression of several hair follicle and epidermal stem cell markers in MCC and found LRIG1 to be a positive prognostic marker in MCC.

**Supplementary Information:**

The online version contains supplementary material available at 10.1007/s00428-021-03158-7.

## Introduction

Merkel cell carcinoma (MCC) is a neuroendocrine carcinoma of the skin. The cellular origins of this rare and highly aggressive skin cancer subtype are thus far unknown. Based on protein expression patterns and ultrastructural findings, MCC tumor cells share many similarities with Merkel cells, mechanoreceptive cells located in the basal layer of the epidermis. In the majority of MCCs—approximately 80% of MCC tumors in the northern hemisphere—the DNA genome of Merkel cell polyomavirus (MCPyV) is integrated in the tumor cell genome, and this is considered the causative agent for tumorigenesis in MCPyV infection [1, 2]. We and others have previously shown significant morphologic and clinicopathological differences between MCPyV-positive and MCPyV-negative MCCs [2–6].

Because of their phenotypic similarities, MCC was initially believed to arise from Merkel cells that had undergone malignant transformation. Several arguments against this theory have since been presented, notably that Merkel cells are post-mitotic and that neuroendocrine carcinomas in other organs tend to arise from epithelial progenitors rather than from the neuroendocrine cells themselves [7–9]. Furthermore, neuroendocrine differentiation in Merkel cell progenitors is triggered by ATOH1 expression [9]. ATOH1 has also been found to be expressed in MCC, and, as such, the acquisition of a Merkel cell-like phenotype in MCC could occur during the oncogenic process [10]. Based on the expression of several B-lymphoid lineage markers in MCC, it has been suggested that the cell of origin for MCC could be a pro-/pre- or pre-B cell [11].

Other cells that have been suggested as the cell of origin for MCC are fibroblasts, dermal stem cells, and epithelial stem cells of the skin, notably the stem cells of the hair follicles [12–15]. Arguments have also been tendered for MCC having more than one cell of origin, for example, MCPyV-positive MCC arising from a dermal progenitor and MCPyV-negative MCC arising from an epithelial stem cell of the skin, which would be consistent with MCPyV-negative MCC harboring a high mutational burden and a UV signature that is lacking in MCPyV-positive MCC [16–18]. Another finding alluding to some cases of MCC arising from epithelial stem cells is the presence of so-called combined MCC, in which MCPyV-negative MCC is found in conjunction with another carcinoma component, most commonly eccrine or squamous cell carcinoma (SCC) [19]. In some cases, these represent collision tumors, but in others, there is evidence of clonality between the MCC and SCC components, hinting at an ancestral cell with differentiation potential into both types of cells [20].

In this study, we aimed to shed further light on the possibility that a subset of MCC tumors arise from epithelial stem cells of the skin by examining the expression of cytokeratin-19 (CK19), leucine-rich repeat-containing G-protein coupled receptor 5 (LGR5), and SRY-box transcription factor 9 (SOX9), markers of hair follicle stem cells [21–23], as well as leucine-rich repeats and immunoglobulin-like domains protein 1 (LRIG1), a marker of both hair follicle stem cells and stem cells of the interfollicular epidermis [24, 25], in MCC and normal human skin. We also aimed to elucidate whether there is a correlation between the expression of these markers and tumor MCPyV status or other clinicopathological characteristics or patient survival.

## Patients and methods

The study protocol was approved by the Ethics Committee of Helsinki University Central Hospital. The Ministry of Health and Social Affairs granted permission to collect patient data and the National Authority for Medicolegal Affairs to collect tissue samples.

### Patients, clinical data, and tissue samples

Data on patients diagnosed with MCC in Finland from 1979 to 2018 were obtained from the Finnish Cancer Registry and Helsinki University Hospital files. Clinical details were extracted from hospital records. Formalin-fixed, paraffin-embedded tissue blocks were retrieved from the pathology archives. MCC diagnoses were confirmed in a blinded fashion from our earlier studies according to well-established criteria by two researchers with special expertise in MCC pathology [26].

MCPyV detection from paraffinized tumor blocks was performed in our previous study and is described in detail elsewhere [2]. Briefly, the presence of MCPyV DNA was analyzed from DNA extracted from representative deparaffinized tumor sections. Quantitation of MCPyV DNA was performed using real-time polymerase chain reaction (PCR). The relative DNA sequence copy number for each tissue sample was expressed as a ratio of MCPyV DNA to protein tyrosine phosphatase gamma receptor gene DNA. The sample was considered positive when MCPyV DNA copy number per reference gene was greater than 0.1. MCPyV large T (LT) antigen expression was evaluated as described earlier [27]. Tissue microarray (TMA) blocks with 374 MCC tissue cores corresponding to 168 patients as well as five formalin-fixed paraffin-embedded tissue blocks containing normal human skin were used for immunohistochemistry.

### Immunohistochemistry

Four-micrometer sections were cut from the TMA blocks to create four slides from each TMA block to be used for immunohistochemical staining of CK19, LGR5, LRIG1, and SOX9. The same was done for the blocks containing normal human skin.

The primary antibodies used were recombinant anti-cytokeratin 19 antibody (clone EP1580Y) (cat# ab52625 Abcam, Cambridge, UK) at 1:1000 dilution, recombinant anti-LGR5 antibody (clone EPR3065Y) (cat# ab75850 Abcam) at 1:150 dilution, anti-LRIG1 antibody (polyclonal) (cat# ab197985 Abcam) at 1:200 dilution, and recombinant anti-SOX9 antibody (clone EPR14335-78) (cat# ab185966 Abcam) at 1:1000 dilution. For all primary antibodies, overnight incubation at a temperature of 4 °C was performed. Primary antibodies were detected by using an Orion detection system rabbit HRP (ready-to-use) kit (WellMed, Duiven, the Netherlands) and an ImmPACT DAB Substrate Kit (Vector Laboratories, Burlingame, CA, USA). Counterstaining was performed with Mayer's hematoxylin (Lillie's modification) (Dako, Carpinteria, CA, USA), and the slides were cover-slipped with Menzel™ Microscope Coverslips.

### Immunostaining evaluation

The TMA slides were digitally scanned using a 3DHISTECH Pannoramic 250 slide scanner, and the TMA spots were examined and evaluated for expression of CK19, LGR5, LRIG1, and SOX9 in their digital form. For the expression of LGR5 and LRIG1, a grading system was used in which the spots were classified as showing no expression, weak positive expression, or intermediate/strong positive expression. For SOX9, the spots were considered to show positive expression if at least 20% of the tumor cells in the spot stained positively for SOX9. For CK19, the spots were classified as showing either dot-like expression or homogeneous expression depending on which expression pattern was present in over 50% of the tumor cells; if no expression was observed, the spot was naturally classified as showing no expression. The degree of immunohistochemical expression for each patient case was determined by the highest degree of expression observed in a TMA spot corresponding to that patient, with homogeneous CK19 expression being considered a higher degree of expression than dot-like expression.

### Statistical analysis

Statistical analysis was performed with SPSS statistics 26.0 software (IBM Corporation, New York, NY, USA). *P* values of less than 0.05 were considered significant. Immunoexpression of CK19, LGR5, LRIG1, and SOX9 was compared with gender, tumor location, sun-exposure pattern, presence of metastasis at diagnosis, MCPyV status, MCPyV LT expression, and immunoexpression of the three other studied markers by χ2 test or Fisher’s exact test. In terms of sun-exposure pattern, the head and neck and limb regions were considered sun-exposed, whereas the trunk was considered sun-protected. MCPyV status was defined as the presence or absence of MCPyV DNA. The statistical associations between the immunoexpression of CK19, LGR5, LRIG1, and SOX9 and the age at time of diagnosis as well as the size of the primary tumor were evaluated by Mann–Whitney analysis or Kruskal–Wallis analysis. Cumulative survival was estimated with the Kaplan-Meyer method, and survival between groups was compared by log-rank (Mantel-Cox) test. Overall survival was calculated from the date of diagnosis to death, censoring subjects alive on their last follow-up date. MCC-specific survival was calculated from the date of diagnosis to death considered to be due to MCC. LRIG1 expression, age, stage at diagnosis, and MCPyV LT expression were considered in a Cox regression multivariate analysis of overall and MCC-specific survival using a subcohort of 90 patients that excluded patients for whom stage at diagnosis was not known, following an exclusion of the two patients known to have stage IV MCC at diagnosis. The staging system employed was the American Joint Committee on Cancer classification for Merkel cell carcinoma, eighth edition.

## Results

### Overview of patients

  Detailed patient clinical data are shown in Table 1. After application of the exclusion criteria (expression data for at least three of the four studied markers and survival data available), our cohort included 137 MCC patients with a mean age at the time of MCC diagnosis of 77 years. Ninety-five (69%) of the patients were female. Two patients (1.5%) were kidney transplant recipients. The most common location for the primary tumors was the head and neck region, affecting 69 cases (55%). In four cases (2.9%), there was no known primary tumor. Tumor MCPyV status was available for 107 patients, with 68% of them being MCPyV-positive. The original cohort size was 168 patients; 27 patients were excluded because they lacked expression data for more than one of the studied markers since some of the TMA spots were missing or unrepresentative and 4 patients were excluded because survival data were missing due to the diagnosis date being unknown.

**Table 1:** Clinicopathological features of patients

Characteristic	*N* = 137 (%)
Sex	
Male	42 (31)
Female	95 (69)
Age	Range 27–100
≤ 50 years	5 (3.6)
51–69 years	23 (17)
70–84 years	68 (50)
85–100 years	41 (30)
Hematological malignancy	
CLL	6 (4.4)
NHL	1 (0.73)
Organ transplant	
Kidney	2 (1.5)
Tumor location	
Head and neck	69 (50)
Torso	14 (10)
Limbs	50 (36)
Unknown primary	4 (2.9)
Stage* at diagnosis (*N* = 92)	
I	46 (34)
II	28 (30)
III	16 (17)
IV	2 (2.2)
Disease progression (*N* = 115)	
Metastasis at diagnosis	20 (17)
MCPyV status (*N* = 107)	
Negative	34 (32)
Positive	73 (68)

^*^American Joint Committee on Cancer classification for Merkel cell carcinoma, eighth edition

*CLL* chronic lymphocytic leukemia, *NHL* non-Hodgkin lymphoma, *MCPyV* Merkel cell polyomavirus

### CK19, LGR5, LRIG1, and SOX9 expression in normal human skin

**Fig. 1:**
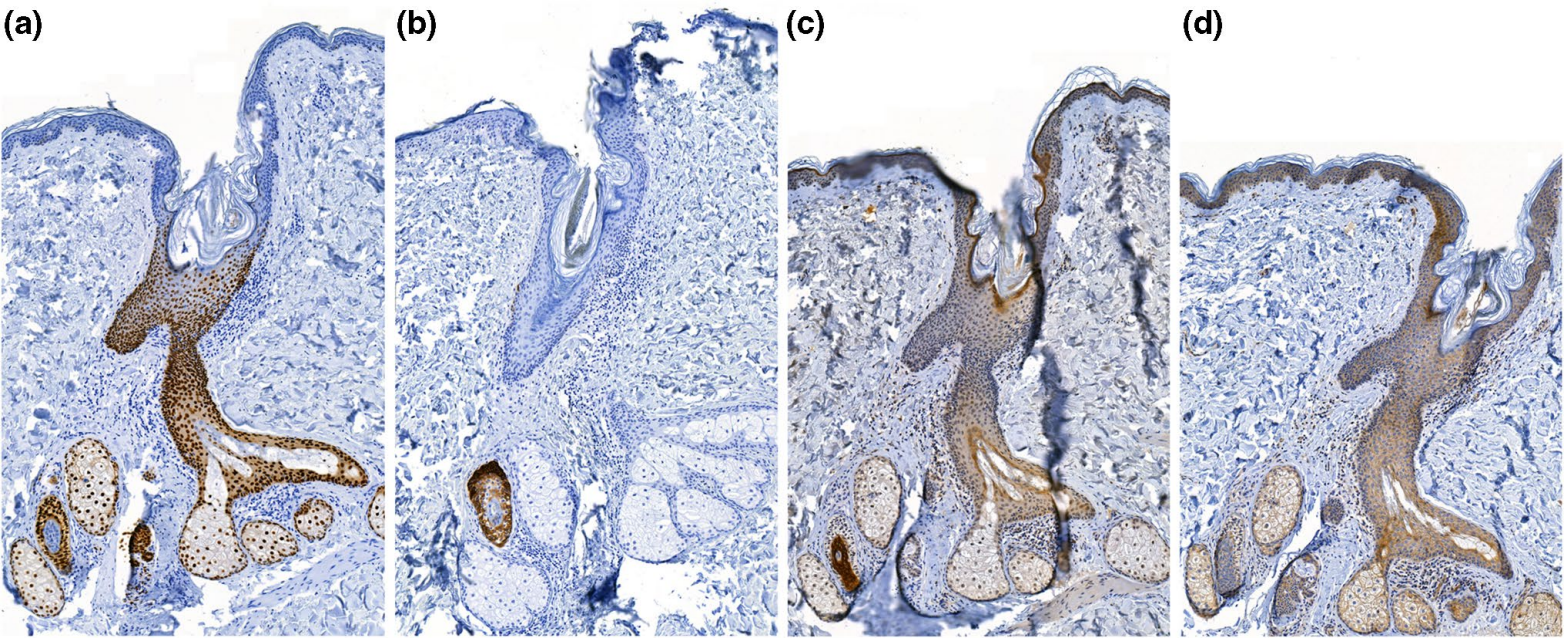
Examples of staining patterns in a hair follicle of normal human skin stained for SOX9 (**a**), CK19 (**b**), LRIG1 (**c**), and LGR5 (**d**)

Demonstrations of staining patterns in normal human skin are shown in Fig. 1. For SOX9, expression in the interfollicular epidermis was generally restricted to individual cells in the basal layer. Expression was also observed in hair follicles as well as sweat- and sebaceous glands. For CK19, expression in the interfollicular epidermis was also generally restricted to individual cells in the basal layer. Expression was also observed in the outer root sheet of hair follicles as well as sweat, but not sebaceous glands. For LRIG1, there was intermediate expression in the hair follicles and interfollicular epidermis, weak expression in sebaceous glands, and strong expression in sweat glands and deeper regions of the hair follicles. For LGR5, expression was quite uniform throughout the interfollicular epidermis and hair follicles as well as sweat and sebaceous glands.

### CK19, LGR5, LRIG1, and SOX9 expression in MCC

**Fig. 2:**
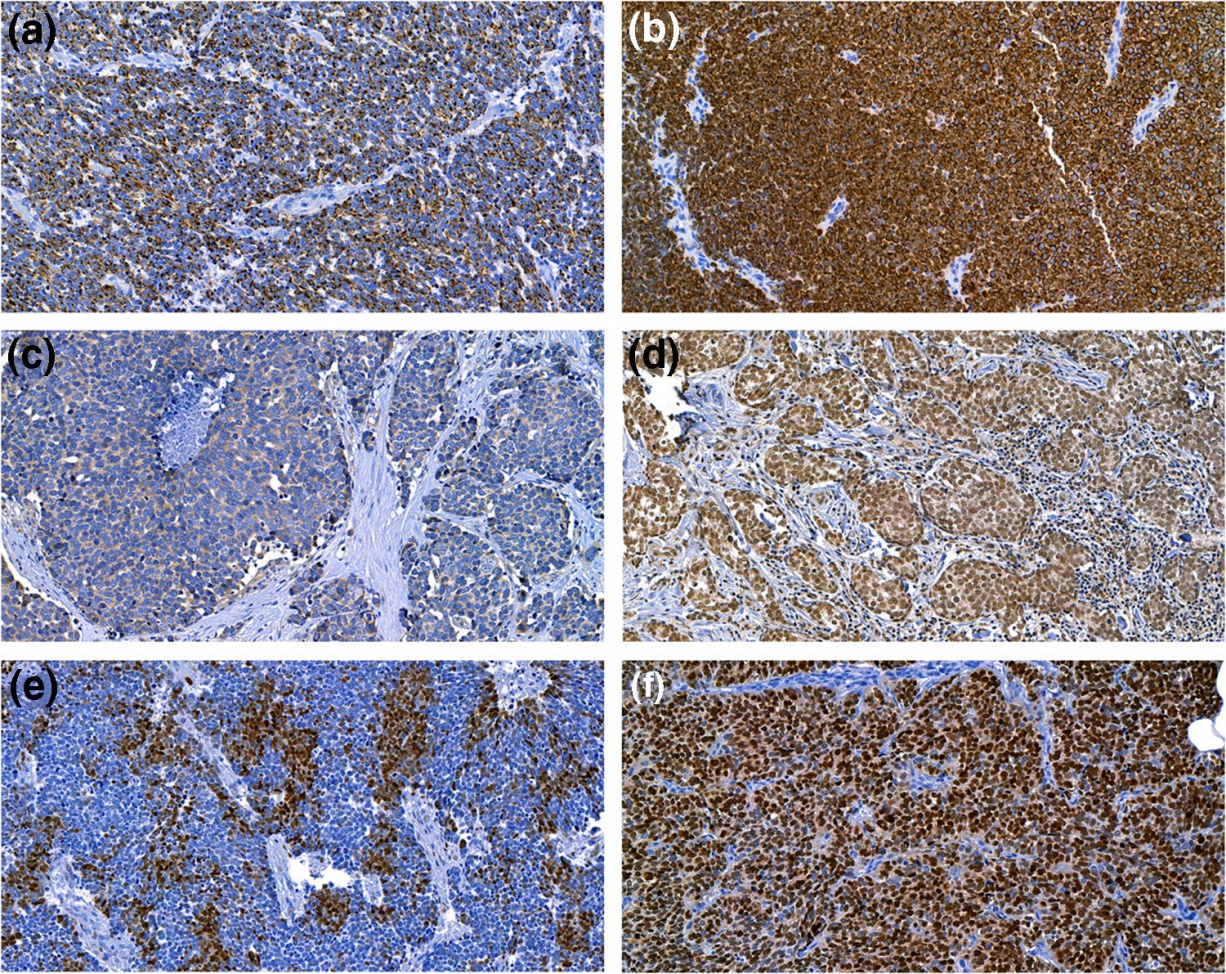
Examples of immunohistochemical staining results depicting dot-like and homogeneously positive CK19 expression (**a** and **b**), intermediate LGR5 expression (**c**), and strong LRIG1 expression (**d**), as well as heterogeneous and more homogeneous SOX9 expression (**e** and **f**)

Demonstrations of the different expression patterns and intensities are shown in Fig. 2, and a summary of the expression rates of the studied markers is provided in Table 2. Detailed data on the correlations between the expression of the studied markers and patient clinicopathological features can be found in Table 3 and Online Resources 1, 2, and 3. For SOX9, we recorded positive immunostaining in 21 samples (15.3%). We observed a significant association between SOX9 expression and MCPyV-negativity (*P* < 0.001) and also between SOX9 expression and head and neck localization of the primary tumor (*P* = 0.039). Furthermore, we noted a significant association between SOX9 expression and homogeneous CK19 expression (*P* = 0.044).

**Table 2:** Summary of epidermal stem cell marker expression rates

	Absent no. (%)	Present no. (%)	
SOX9	116 (84.7)	21 (16.1)	
	Absent no. (%)	Dot-like no. (%)	Homogenous no. (%)
CK19	3 (2.2)	30 (22.1)	103 (75.7)
	Absent no. (%)	Weak no. (%)	Intermediate/strong no. (%)
LGR5	19 (13.9)	42 (30.7)	76 (55.5)
LRIG1	19 (14.0)	62 (45.6)	55 (40.4)

**Table 3:** Patient and tumor characteristics according to LRIG1 expression

LRIG1 expression	Absent (*n* = 19) no. (%)	Weak (*n* = 62) no. (%)	Inter/strong (*n* = 55) no. (%)	*P* value
Variable				
MCPyV DNA				
Absent (< 0.1 copies)	9 (56.3)	14 (25.5)	11 (31.4)	0.067
Present (≥ 0.1 copies)	7 (43.7)	41 (74.5)	24 (68.6)	
N.A	3	7	20	
MCPyV LT expression				
Absent	11 (68.8)	18 (32.1)	15 (32.6)	0.020
Present	5 (31.2)	38 (67.9)	31 (67.4)	
N.A	3	6	9	
Gender				
Female	11 (57.9)	51 (82.3)	32 (58.2)	0.010
Male	8 (42.1)	11 (17.7)	23 (41.8)	
Tumor site				
Head or neck	14 (73.7)	31 (50.8)	24 (46.2)	0.268
Trunk	2 (10.5)	7 (11.5)	5 (9.6)	
Limb	3 (15.8)	23 (37.7)	23 (44.2)	
Unknown primary	0	1	3	
Sun exposure				
Sun-exposed	17 (90.5)	54 (88.5)	47 (90.4)	0.950
Sun-protected	2 (10.5)	7 (11.5)	5 (9.6)	
Unknown primary	0	1	3	
Metastasis at diagnosis				
Absent	12 (85.7)	42 (82.4)	40 (81.6)	0.939
Present	2 (14.3)	9 (17.6)	9 (18.4)	
N.A	5	11	6	
Age at diagnosis, y				
Median (range)	85 (50–100)	80.5 (47–93)	76.0 (27–93)	0.003
Tumor diameter, mm				
Median (range)	15.0 (10–30)	16.0 (6–75)	15.5 (5–85)	0.886
N.A	10	19	17	

We observed expression of CK19 in 133 samples (97.8%). Homogeneous expression was seen in 103 (75.7%) and dot-like expression in 30 (22.1%) of the samples. Dot-like expression of CK19 was significantly associated with MCPyV-positivity (*P* = 0.034).

For LGR5, we observed positive immunohistochemical staining in 118 samples (86.1%). Intermediate or strong expression was detected in 76 (55.5%) and weak expression in 42 samples (30.7%). There was a significant association between the degree of LGR5 expression and sun-exposed localization of the primary tumor that was specifically caused by preferential sun-exposed localization of cases exhibiting intermediate or strong LGR5 expression (*P* = 0.024).

**Fig. 3:**
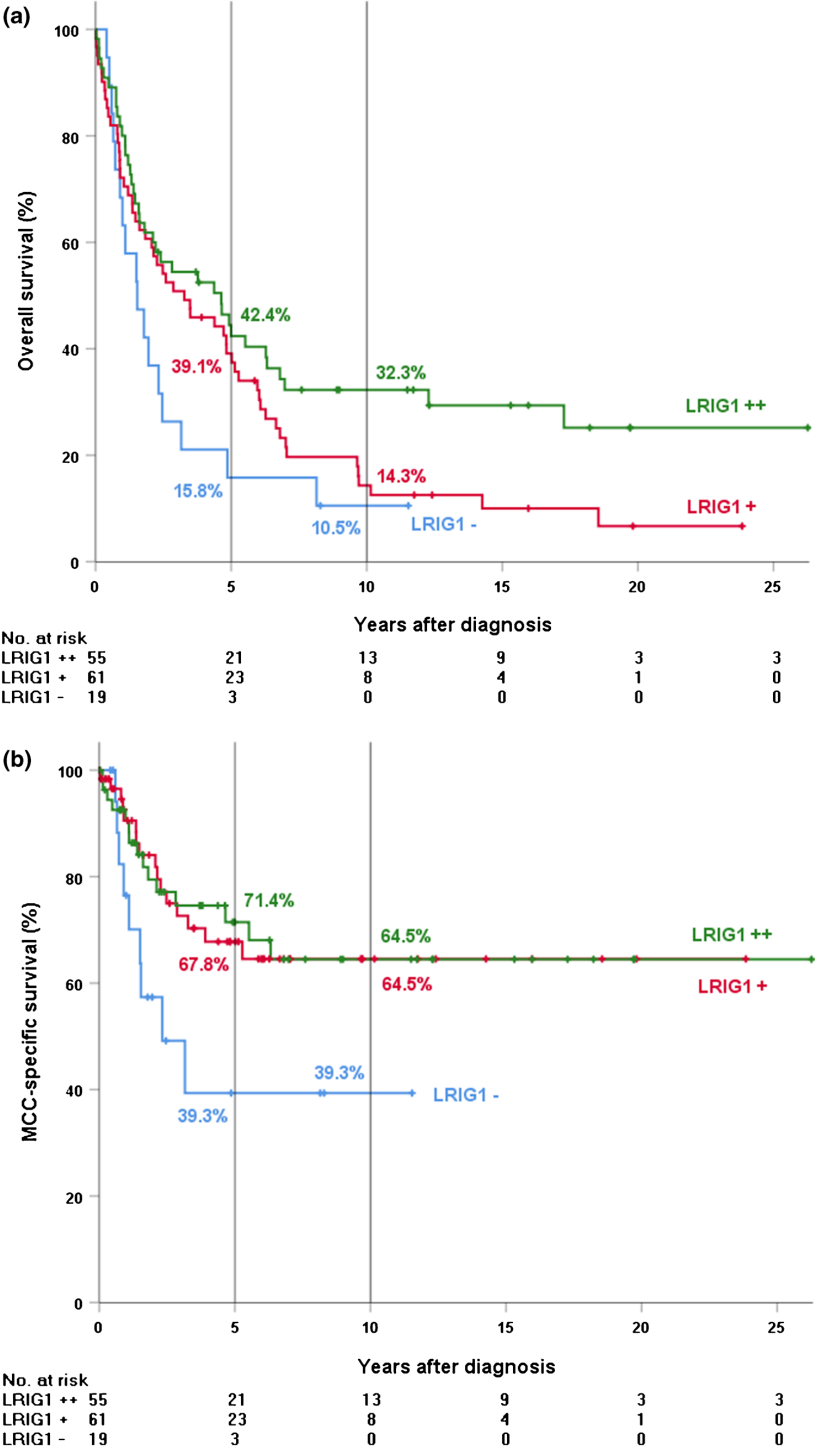
Kaplan–Meier analysis of survival among patients whose tumors exhibited intermediate or strong (+ +), weak ( +), or negative (-) LRIG1 expression. **a** Overall survival. **b** Merkel cell carcinoma (MCC)-specific survival. Subject whose tumor exhibited LRIG1 expression had better overall survival (hazard ratio [HR] of death for weak expression = 0.72, 95% confidence interval [CI] = 0.42 to 1.24, and HR of death for intermediate or strong expression = 0.49, 95% CI = 0.28 to 0.88, *P* = 0.037) and MCC-specific survival (HR of death for weak expression = 0.43, 95% CI = 0.19 to 0.98, and HR of death for intermediate or strong expression = 0.42, 95% CI = 0.18 to 0.97, *P* = 0.021*) than those whose tumor lacked LRIG1 expression. Five- and 10-year survival data are shown. *Obtained by comparing cases exhibiting no LRIG1 expression to cases exhibiting any degree of positive LRIG1 expression

Positive staining for LRIG1 was observed in 117 samples (86.0%; Table 3). Intermediate or strong expression was observed in 55 (40.4%) and weak expression in 42 samples (45.6%). We found a significant association between a higher degree of LRIG1 expression and better overall survival (*P* = 0.037; Fig. 3). The 5-year overall survival rate was 15.8% for LRIG1-negative cases, 39.1% for cases showing weak LRIG1 expression, and 42.2% for cases showing intermediate or strong LRIG1 expression. A significant association also emerged between any degree of positive LRIG1 expression and better MCC-specific survival (*P* = 0.021). The 5-year MCC-specific survival rate was 39.3% for LRIG1-negative cases and 69.5% for cases exhibiting any degree of positive LRIG1 expression. We also noted a significant association between a higher degree of LRIG1 expression and younger age at time of diagnosis (*P* = 0.003) and MCPyV LT expression (*P* = 0.020). For LRIG1-negative cases, the median age at diagnosis and the expression rate of MCPyV LT were 85 years and 68.8%, respectively. For cases with weak LRIG1 expression, the corresponding numbers were 80.5 years and 32.1%, and for cases with intermediate or strong LRIG1 expression 76 years and 32.6%.

To examine whether LRIG1 was an independent prognostic factor, univariate analyses of overall survival were carried out for age, gender, stage at diagnosis, MCPyV LT expression, and MCPyV DNA status to identify prognostic factors to include in a multivariate analysis together with LRIG1. Of these factors, younger age (*P* < 0.001), lower stage at diagnosis (*P* < 0.001), and MCPyV LT expression (*P* = 0.001) were associated with better prognosis, but no prognostic significance was found for MCPyV DNA status (*P* = 0.064) or gender (*P* = 0.691). In Cox multivariate analysis (Table 4), for both overall and MCC-specific survival, the factors that exhibited independent statistical significance were age, stage at diagnosis, and MCPyV LT expression (all *P* values ≤ 0.012).

**Table 4:** Cox regression multivariate analysis of overall and MCC-specific survival

Overall survival		
	HR (95% CI)	*P* value
Age	1.07 (1.04–1.10)	< 0.001
MCPyV LT expression	0.41 (0.23–0.74)	0.003
LRIG1 expression		
Absent	1.00	
Weak	0.69 (0.30–1.56)	0.369
Intermediate/strong	0.78 (0.32–1.91)	0.589
Stage at diagnosis		
I	1.00	
II	1.18 (0.66–2.12)	0.578
III	3.79 (1.76–8.16)	0.001
MCC-specific survival		
	HR (95% CI)	*P* value
Age	1.06 (1.01–1.11)	0.012
MCPyV LT expression	0.15 (0.05–0.44)	< 0.001
LRIG1 expression		
Absent	1.00	
Weak	0.40 (0.09–1.73)	0.222
Intermediate/strong	0.73 (0.16–3.35)	0.686
Stage at diagnosis		
I	1.00	
II	6.14 (1.81–20.82)	0.004
III	22.98 (5.99–88.17)	< 0.001

*HR* hazard ratio, *CI* confidence interval

For CK19, LGR5, and SOX9, no correlation existed between the degree or pattern of expression and patient survival.

## Discussion

We found frequent expression of epithelial stem cell markers in MCC. Of note, human Merkel cells have been found to express LRIG1, and SOX9 expression has been observed in Merkel cell progenitors in mice [28, 29]. As such, the expression of epithelial stem cell markers in both MCC and Merkel cells and their progenitors could be explained either by a common ancestor or by MCC indeed developing from Merkel cells. Another possibility that should be entertained is that the expression of epithelial stem cell markers could be induced by MCPyV or otherwise acquired during the oncogenic process, thus mimicking an epithelial stem cell expression pattern. That this is quite a realistic scenario in another context was recently demonstrated by Park et al. who found that MCPyV small T antigen (sTag) was able to turn on the expression of INSM1, a marker of neuroendocrine differentiation, in MCC. Crucially, this INSM1 expression was induced by MCPyV sTag and not by ASCL1 or Phox2b, neither of which are expressed in MCC, as is usually the case in neuroendocrine cells under physiological circumstances [30].

It has been a matter of debate whether human Merkel cells are derived from the neural crest or are of epidermal lineage. Murine Merkel cells have been demonstrated to be of epidermal origin [31], and there is substantial evidence suggesting that this is the case in humans as well, including evidence of intraepidermal formation of Merkel cells in xenografts of human fetal skin [28, 32]. The population of Merkel cell progenitors in humans has yet to be thoroughly characterized, and thus, our knowledge of these cells is mainly based on studies done in mice. In mice, stem cells bearing Merkel cell differentiation potential are mainly located in the bulge region and outer root sheet of the hair follicle as well as in touch domes of the interfollicular epidermis [12, 33, 34]. These hair follicle and touch dome stem cells have been found to be the preferential cells of origin in basal cell carcinoma [35]. Basal cell carcinoma has also been shown to express the epithelial stem cell markers CK19, LGR5, LRIG1, and SOX9 [36, 37]. The natural potential of these epithelial stem cells to differentiate into Merkel cells, their sensitivity to oncogenic stimuli such as UV radiation, and the fact that MCC exhibits expression of epithelial stem cell markers known to be expressed in another malignancy that originates from these cells advocates the possibility that they may serve as cells of origin in MCC as well.

SOX9 expression and intermediate or strong LGR5 expression were preferentially observed in tumors of sun-exposed localization, and SOX9 expression was associated with the MCPyV-negative subtype. Assuming that MCC can have both an epidermal and a dermal origin as suggested by Sunshine et al. [16], it would be logical to expect tumors of epidermal origin to preferentially arise in sun-exposed locations and be of the MCPyV-negative subtype, seeing as UV radiation is an important oncogenic factor in epidermal malignancies and MCPyV-negative MCC. Alternatively, seeing as SOX9 expression was associated with the MCPyV-negative subtype and LRIG1 expression was associated with the MCPyV-positive subtype, one could speculate that MCPyV-positive and MCPyV-negative MCC has two distinct epithelial ancestries. Acting on the assumption that MCC generally has an epithelial origin, Kervarrec et al. recently suggested the possibility that UV-induced MCC derives from a keratinocytic progenitor from the interfollicular epidermis that acquires the ability to differentiate into Merkel cells during the oncogenic process, whereas MCPyV-driven oncogenesis is initiated in a progenitor from a hair follicle [17].

The expression of SOX9 in MCC was recently studied by Kervarrec et al., and nuclear SOX9 expression was found to be more frequent in MCPyV-negative cases, as was the case in this study as well [38]. Dot-like cytoplasmic expression of SOX9 was found in 64%, and nuclear expression was found in 28% of the 103 cases. Contrary to these findings, we only observed nuclear expression of SOX9, representing the active form of this transcription factor, but no dot-like cytoplasmic expression. Of note, different antibody clones for SOX9 were used for the two studies.

We observed that dot-like expression of CK19 was more frequent in MCPyV-positive cases. Kervarrec et al. recently found dot-like expression of cytokeratins 8, 18, and 20 to be more frequent in MCPyV-positive MCC [39], and Verhaegen et al. were previously able to induce dot-like expression of cytokeratins 8 and 20 upon ectopic expression of MCPyV sTag in Merkel cells in a transgenic mouse model [40]. Together, these results are suggestive of the capability of MCPyV T antigens to disrupt cytoskeletal organization.

We observed that a higher degree of LRIG1 expression was associated with a better overall survival and that any degree of positive LRIG1 expression was associated with a better MCC-specific survival; however, it was not an independent prognostic marker in Cox regression multivariate analyses when age, stage at diagnosis, and MCPyV LT expression were taken into account. We also found a higher degree of LRIG1 expression to be associated with MCPyV LT expression. As such, it is likely that the result of the univariate analysis was mainly due to the association between LRIG1 expression and MCPyV-positive status. It should be mentioned, however, that in the subcohort of 90 patients used for multivariate analyses, there were only eight cases in which LRIG1 expression was absent. LRIG1 is a known tumor suppressor and has been shown to be a positive prognostic marker in other malignancies such as hepatocellular carcinoma [41–43]. LRIG1 is a membrane protein with a transmembrane domain and an extracellular domain. The extracellular domain has receptor tyrosine kinase (RTK) inhibitory activity, thus suppressing tumor growth by blocking RTK signaling. The ectodomain of LRIG1 has been used as a soluble compound in patient-derived glioblastoma models in vivo with promising results, and the drug resveratrol has been found to inhibit glioma cell growth and promote its apoptosis by upregulating LRIG1 gene expression [44, 45].

Interestingly, LRIG1 expression has been found to be associated with favorable prognosis and presence of human papillomavirus DNA in both oropharyngeal cancer and cervical adenocarcinoma. These represent two other examples of a virus-associated malignancy, in which the virus-positive subtype has a better prognosis. In oropharyngeal cancer, with a sample size of 278 patients, LRIG1 expression was also reported to be an independent prognostic marker in Cox regression multivariate analysis, whereas in cervical adenocarcinoma (sample size 86 patients), it was not [46, 47].

Seeing as MCPyV status was a statistically more relevant prognostic factor than LRIG1 and there was a correlation between LRIG1- and MCPyV-positivity, we consider MCPyV status to be a more suitable prognostic marker for clinical use and the use of both markers for prognostic purposes to be redundant.

In order to prove that a subset of MCC tumors arise from epithelial stem cells, large data sets of for example whole genome sequencing or lineage tracing experiments are necessary. Using large gene expression and methylation data sets, Chteinberg et al. recently found MCC DNA methylation age to be significantly lower than chronological age, indicating a certain degree of stemness of MCC cells; they did not, however, find evidence for pluripotency of MCC cells. [48]

It should be noted that there is limited knowledge of the expression of the studied markers in human skin, as most studies to date have been done in mice. Of note, however, in a study by Quist et al., no expression of LGR5 was observed in the interfollicular epidermis, and expression of LRIG1 was limited to the stratum basale, whereas we found expression of both markers throughout the interfollicular epidermis [35]. Indeed, the expression of LGR5 and LRIG1 that we observed in normal human skin was surprisingly widespread. There are, however, numerous examples of stem cell markers that are also commonly expressed in various normal tissues and tissue specificity might vary with antibody clone [49].

In summary, we found that MCC expresses several epithelial stem cell markers and that LRIG1 is a positive prognostic marker in MCC.

## Supplementary Information

Below is the link to the electronic supplementary material.

Supplementary file1 (DOCX 16 KB)

Supplementary file2 (DOCX 15 KB)

Supplementary file3 (DOCX 16 KB)

## Data Availability

Not applicable.
