# Supplementary material for: LRIG1 is a positive prognostic marker in Merkel cell carcinoma and Merkel cell carcinoma expresses epithelial stem cell markers
Source: Virchows Arch. 2021 Jul 31;479(6):1197–207. doi: 10.1007/s00428-021-03158-7 (PMC8724115; doi:10.1007/s00428-021-03158-7)
Supplement: Supplementary file 1 — Supplementary file1 (DOCX 16 KB) [file 428_2021_3158_MOESM1_ESM.docx]

| **Online Resource 1** Patient and tumor characteristics according to SOX9 expression. | | | |
| --- | --- | --- | --- |
| **SOX9 expression** | **Absent** (n=116) No. (%) | **Present** (n=21) No. (%) | **P-value** |
| **Variable** |  |  |  |
| **MCPyV DNA** |  |  |  |
| Absent (<0.1 copies) | 19 (20.9) | 15 (93.8) | <0.001 |
| Present (≥0.1 copies) | 72 (79.1) | 1 (6.2) |  |
| N.A. | 25 | 5 |  |
| **MCPyV LT expression** |  |  |  |
| Absent | 25 (25.3) | 19 (95.0) | <0.001 |
| Present | 74 (74.7) | 1 (5.0) |  |
| N.A. | 17 | 1 |  |
| **Gender** |  |  |  |
| Female | 84 (72.4) | 11 (52.4) | 0.067 |
| Male | 32 (27.6) | 10 (47.6) |  |
| **Tumor site** |  |  |  |
| Head or neck | 53 (47.3) | 16 (76.2) | 0.039 |
| Trunk | 12 (10.7) | 2 (9.5) |  |
| Limb | 47 (42.0) | 3 (14.3) |  |
| Unknown primary | 4 | 0 |  |
| **Sun-exposure** |  |  |  |
| Sun-exposed | 100 (89.3) | 19 (90.5) | 1.000 |
| Sun-protected | 12 (10.7) | 2 (9.5) |  |
| Unknown primary | 4 | 0 |  |
| **Metastasis at diagnosis** |  |  |  |
| Absent | 81 (82.7) | 14 (82.4) | 1.000 |
| Present | 17 (17.3) | 3 (17.6) |  |
| N.A. | 18 | 4 |  |
| **Age at diagnosis, y** |  |  |  |
| Median (range) | 79.0 (27-93) | 83.0 (65-100) | 0.052 |
| **Tumor diameter, mm** |  |  |  |
| Median (range) | 16.5 (6-85) | 11.5 (5-30) | 0.121 |
| N.A. | 38 | 9 |  |
| **CK19 expression** |  |  |  |
| Dot-like | 29 (25.9) | 1 (4.8) | 0.044 |
| Homogenous | 83 (74.1) | 20 (95.2) |  |
| N.A. | 4 | 0 |  |
| **LGR5 expression** |  |  |  |
| Absent | 18 (15.5) | 1 (4.8) | 0.422 |
| Weak | 35 (30.2) | 7 (33.3) |  |
| Intermediate/strong | 63 (54.3) | 13 (61.9) |  |
| **LRIG1 expression** |  |  |  |
| Absent | 14 (12.2) | 5 (23.8) | 0.355 |
| Weak | 53 (46.1) | 9 (42.9) |  |
| Intermediate/strong | 48 (41.7) | 7 (33.3) |  |
| N.A. | 1 | 0 |  |

**Article title:** LRIG1 is a Positive Prognostic Marker in Merkel Cell Carcinoma and Merkel Cell Carcinoma Expresses Epithelial Stem Cell Markers

**Journal name:** Virchow Archiv: European Journal of Pathology

**Author names:** Benjamin Sundqvist, Harri Sihto, Maria von Willebrand, Tom Böhling, Virve Koljonen

**Affiliation and e-mail address of the corresponding author:** Benjamin Sundqvist, Department of Pathology, University of Helsinki, Helsinki, Finland, benjamin.sundqvist@helsinki.fi
