# Supplementary material for: LRIG1 is a positive prognostic marker in Merkel cell carcinoma and Merkel cell carcinoma expresses epithelial stem cell markers
Source: Virchows Arch. 2021 Jul 31;479(6):1197–207. doi: 10.1007/s00428-021-03158-7 (PMC8724115; doi:10.1007/s00428-021-03158-7)
Supplement: Supplementary file 2 — Supplementary file2 (DOCX 15 KB) [file 428_2021_3158_MOESM2_ESM.docx]

| **Online Resource 2** Patient and tumor characteristics according to CK19 expression. | | | |
| --- | --- | --- | --- |
| **CK19 expression** | **Dot-like** (n=30) No. (%) | **Homogenous** (n=103) No. (%) | **P-value** |
| **Variable** |  |  |  |
| **MCPyV DNA** |  |  |  |
| Absent (<0.1 copies) | 3 (13.0) | 29 (36.3) | 0.034 |
| Present (≥0.1 copies) | 20 (83.0) | 51 (63.7) |  |
| N.A. | 7 | 23 |  |
| **MCPyV LT expression** |  |  |  |
| Absent | 4 (15.4) | 38 (42.7) | 0.011 |
| Present | 22 (84.6) | 51 (67.3) |  |
| N.A. | 4 | 14 |  |
| **Gender** |  |  |  |
| Female | 24 (80.0) | 68 (66.0) | 0.144 |
| Male | 6 (20.0) | 35 (34.0) |  |
| **Tumor site** |  |  |  |
| Head or neck | 15 (53.6) | 53 (52.5) | 0.937 |
| Trunk | 3 (10.7) | 9 (8.9) |  |
| Limb | 10 (35.7) | 39 (38.6) |  |
| Unknown primary | 2 | 2 |  |
| **Sun-exposure** |  |  |  |
| Sun-exposed | 25 (89.3) | 92 (91.1) | 0.722 |
| Sun-protected | 3 (10.7) | 9 (8.9) |  |
| Unknown primary | 2 | 2 |  |
| **Metastasis at diagnosis** |  |  |  |
| Absent | 20 (80.0) | 73 (84.9) | 0.548 |
| Present | 5 (20.0) | 13 (15.1) |  |
| N.A. | 5 | 17 |  |
| **Age at diagnosis, y** |  |  |  |
| Median (range) | 74.5 (47-93) | 80.0 (27-100) | 0.152 |
| **Tumor diameter, mm** |  |  |  |
| Median (range) | 18.0 (7-40) | 15.0 (5-85) | 1.000 |
| N.A. | 10 | 36 |  |
| **LGR5 expression** |  |  |  |
| Absent | 4 (13.3) | 14 (13.6) | 0.996 |
| Weak | 9 (30.0) | 30 (29.1) |  |
| Intermediate/strong | 17 (56.7) | 59 (57.3) |  |
| **LRIG1 expression** |  |  |  |
| Absent | 2 (6.7) | 16 (15.7) | 0.131 |
| Weak | 18 (60.0) | 41 (40.2) |  |
| Intermediate/strong | 10 (33.3) | 45 (44.1) |  |
| N.A. | 0 | 1 |  |

**Article title:** LRIG1 is a Positive Prognostic Marker in Merkel Cell Carcinoma and Merkel Cell Carcinoma Expresses Epithelial Stem Cell Markers

**Journal name:** Virchow Archiv: European Journal of Pathology

**Author names:** Benjamin Sundqvist, Harri Sihto, Maria von Willebrand, Tom Böhling, Virve Koljonen

**Affiliation and e-mail address of the corresponding author:** Benjamin Sundqvist, Department of Pathology, University of Helsinki, Helsinki, Finland, benjamin.sundqvist@helsinki.fi
