# Supplementary material for: LRIG1 is a positive prognostic marker in Merkel cell carcinoma and Merkel cell carcinoma expresses epithelial stem cell markers
Source: Virchows Arch. 2021 Jul 31;479(6):1197–207. doi: 10.1007/s00428-021-03158-7 (PMC8724115; doi:10.1007/s00428-021-03158-7)
Supplement: Supplementary file 3 — Supplementary file3 (DOCX 16 KB) [file 428_2021_3158_MOESM3_ESM.docx]

| **Online Resource 3** Patient and tumor characteristics according to LGR5 expression. | | | |  |
| --- | --- | --- | --- | --- |
| **LGR5 expression** | **Absent** (n=19) No. (%) | **Weak** (n=42) No. (%) | **Inter/strong** (n=76) No. (%) | **P- value** |
| **Variable** |  |  |  |  |
| **MCPyV DNA** |  |  |  |  |
| Absent (<0.1 copies) | 6 (33.3) | 12 (34.3) | 16 (29.6) | 0.888 |
| Present (≥0.1 copies) | 12 (66.7) | 23 (65.7) | 38 (70.4) |  |
| N.A. | 1 | 7 | 22 |  |
| **MCPyV LT expression** |  |  |  |  |
| Absent | 7 (43.8) | 13 (35.1) | 24 (36.4) | 0.827 |
| Present | 9 (56.2) | 24 (64.9) | 42 (63.6) |  |
| N.A. | 3 | 5 | 10 |  |
| **Gender** |  |  |  |  |
| Female | 11 (57.9) | 29 (69.0) | 55 (72.4) | 0.472 |
| Male | 8 (42.1) | 13 (31.0) | 21 (17.6) |  |
| **Tumor site** |  |  |  |  |
| Head or neck | 12 (63.2) | 23 (56.1) | 34 (46.6) | 0.009 |
| Trunk | 3 (15.8) | 8 (19.5) | 3 (4.1) |  |
| Limb | 4 (21.0) | 10 (24.4) | 36 (49.3) |  |
| Unknown primary | 0 | 1 | 3 |  |
| **Sun-exposure** |  |  |  |  |
| Sun-exposed | 16 (84.2) | 33 (80.5) | 70 (95.9) | 0.024 |
| Sun-protected | 3 (15.8) | 8 (19.5) | 3 (4.1) |  |
| Unknown primary | 0 | 1 | 3 |  |
| **Metastasis at diagnosis** |  |  |  |  |
| Absent | 17 (94.4) | 30 (88.2) | 48 (76.2) | 0.116 |
| Present | 1 (5.6) | 4 (11.8) | 15 (23.8) |  |
| N.A. | 1 | 8 | 13 |  |
| **Age at diagnosis, y** |  |  |  |  |
| Median (range) | 79.0 (35-100) | 77.0 (47-92) | 80.5 (27-93) | 0.707 |
| **Tumor diameter, mm** |  |  |  |  |
| Median (range) | 19.0 (10-50) | 12.0 (5-50) | 16.0 (8-85) | 0.092 |
| N.A. | 3 | 15 | 29 |  |
| **LRIG1 expression** |  |  |  |  |
| Absent | 9 (47.4) | 4 (9.8) | 6 (7.9) | <0.001 |
| Weak | 7 (36.8) | 27 (65.8) | 28 (36.8) |  |
| Intermediate/strong | 3 (15.8) | 10 (24.4) | 42 (55.3) |  |
| N.A. | 0 | 1 | 0 |  |

**Article title:** LRIG1 is a Positive Prognostic Marker in Merkel Cell Carcinoma and Merkel Cell Carcinoma Expresses Epithelial Stem Cell Markers

**Journal name:** Virchow Archiv: European Journal of Pathology

**Author names:** Benjamin Sundqvist, Harri Sihto, Maria von Willebrand, Tom Böhling, Virve Koljonen

**Affiliation and e-mail address of the corresponding author:** Benjamin Sundqvist, Department of Pathology, University of Helsinki, Helsinki, Finland, benjamin.sundqvist@helsinki.fi
